# Disturbed microbial ecology in Alzheimer’s disease: evidence from the gut microbiota and fecal metabolome

**DOI:** 10.1186/s12866-021-02286-z

**Published:** 2021-08-12

**Authors:** Jianxiong Xi, Ding Ding, Huiwei Zhu, Ruru Wang, Feng Su, Wanqing Wu, Zhenxu Xiao, Xiaoniu Liang, Qianhua Zhao, Zhen Hong, Hua Fu, Qianyi Xiao

**Affiliations:** 1 Department of Preventive Medicine and Health Education, School of Public Health, Fudan University, Shanghai, 200032 China; 2 Institute of Neurology, Huashan Hospital, Fudan University, Shanghai, 200040 China; 3 National Clinical Research Center for Aging Diseases, Shanghai, 200040 China

**Keywords:** Alzheimer’s disease, Gut microbiota, Fecal metabolome, Inflammatory cytokines, Fecal markers

## Abstract

**Background:**

Gut microbiota (GMB) alteration has been reported to influence the Alzheimer’s disease (AD) pathogenesis through immune, endocrine, and metabolic pathways. This study aims to investigate metabolic output of the dysbiosis of GMB in AD pathogenesis. In this study, the fecal microbiota and metabolome from 21 AD participants and 44 cognitively normal control participants were measured. Untargeted GMB taxa was analyzed through 16S ribosomal RNA gene profiling based on next-generation sequencing and fecal metabolites were quantified by using ultrahigh performance liquid chromatography-mass spectrometry (UPLC-MS).

**Results:**

Our analysis revealed that AD was characterized by 15 altered gut bacterial genera, of which 46.7% (7/15 general) was significantly associated with a series of metabolite markers. The predicted metabolic profile of altered gut microbial composition included steroid hormone biosynthesis, N-Acyl amino acid metabolism and piperidine metabolism. Moreover, a combination of 2 gut bacterial genera (*Faecalibacterium* and *Pseudomonas*) and 4 metabolites (N-Docosahexaenoyl GABA, 19-Oxoandrost-4-ene-3,17-dione, Trigofoenoside F and 22-Angeloylbarringtogenol C) was able to discriminate AD from NC with AUC of 0.955 in these 65 subjects.

**Conclusions:**

These findings demonstrate that gut microbial alterations and related metabolic output changes may be associated with pathogenesis of AD, and suggest that fecal markers might be used as a non-invasive examination to assist screening and diagnosis of AD.

**Supplementary Information:**

The online version contains supplementary material available at 10.1186/s12866-021-02286-z.

## Background

Alzheimer’s disease (AD) is the most prevalent neurodegenerative disorder and is characterized by extracellular plaques composed of amyloid-β (Aβ) peptide and intracellular neurofibrillary tangles composed of hyperphosphorylated tau protein [1]. Emerging evidences show that dysbiosis and alterations of the intestinal microbiota contribute to the development of neurodegenerative diseases, including Parkinson’ s disease, schizophrenia, stroke, epilepsy and depression via the “microbiota-gut-brain” axis [2–7]. A few studies have revealed the relationship between gut microbiota (GMB) and AD. Decreased diversity in microbiota is reported in AD and mild cognitive impairment (MCI) patients compared with normal controls, and changes in intestinal microbiota could be used for early detection of AD [8–10]. Some populations of enterotype bacteria are differential between demented and non-demented dementia [11]. A recent study shows that the fecal and blood microbiota in MCI patients are similar to that of AD patients compared with normal controls, suggesting the bacterial dysbiosis preceded the onset of dementia [12]. Fecal microbiota transplantation in mice also supports a possible connection between the GMB and AD [13, 14].

The clarification of the gut-brain axis in AD pathogenesis would provide accessible markers from feces. Human intestinal tract harbors a complex community of microbes with the vast majority of the resident microbial population [15], which plays an important role in the maintenance of human health, including energy extraction, vitamin biosynthesis, prevention of pathogen overgrowth and education of the immune system [16]. Several studies suggest that GMB impact brain through a bidirectional communication system that is connected via neural, immune, endocrine, and metabolic pathways [17–20]. Although 16S ribosomal RNA (rRNA) gene sequencing allows for profiling of complex microbial composition and indicating the microbial entities [21], annotation and the actual microbial activity of GMB is sparse as 16S rRNA gene sequence only provides a global composition analysis but cannot distinguish alive and dead microbes [22]. Fecal metabolites, served as the metabolic output of both GMB and cellular metabolism occurring inside the human intestinal tract, could complement sequencing-based approaches with showing functional readout of microbial activity [23]. Recent evidence indicated that the compounds secreted or influenced by bacteria may cause systemic inflammatory reactions, and then damage the blood-brain barrier (BBB) and promote neurodegeneration [3, 24, 25]. A recent study reported that during AD progression, the alteration of GMB composition leads to the peripheral accumulation of phenylalanine and isoleucine and the increase of pro-inflammatory T helper 1 cell frequency in the blood in AD mouse models, which were also observed in MCI due to AD in human [26]. Based on these findings, we sought to investigate potentially AD-relevant gut microbes and their metabolic outputs [27], which has rarely been reported in AD.

Therefore, in this study, we investigated the altered GMB composition and metabolic changes in the fecal samples that are relevant with AD, and examined their correlation by integrating the two omics findings. Based on the correlation analysis, we determined the potentially functional microbiota and its metabolic output, and further identified the potentially fecal markers of AD. Considering that compounds influenced by bacteria may cause systemic inflammatory reactions, and the altered cytokine levels had been reported as potential hallmark of AD, we also analyzed the association between differential fecal markers and peripheral inflammatory cytokines that linked with AD to further understand the function of altered GMB composition.

## Results

### Characteristics of study subjects

The demographic and clinical information for 21 AD participants and 44 cognitively normal control (NC) participants was presented in Table 1. No statistically significant difference in proportion of sex was found (*P* < 0.05) between AD group and NC group. AD and NC groups also did not differ with respect to age at examination, education years and body mass index (BMI) (*P* < 0.05). The mean MMSE score was significantly lower in AD participants (18.0) than in NC participants (29.0), *P* < 0.001. *APOE ε4* carrier rate was significantly higher in AD participants (50.0%) than in NC participants (11.9%), *P* < 0.001.

**Table 1:** Clinical characteristics of AD subjects and cognitively normal control (NC) subjects

Baseline characteristic	AD	NC	*P* value^a^
Participants, n	21	44	
Age, years (IQR)	76.2 (9.9)	78.4 (6.6)	0.262
Female, n (%)	8 (38.1)	24 (54.6)	0.215
Education, years (IQR)	12 (3)	12 (7)	0.889
MMSE scores, mean (IQR)	18 (8)	29 (2)	< 0.001
*APOE* ε4, n (%)	10 (50.0)^b^	5 (11.9)^c^	< 0.001
BMI, mean (IQR)	22.8 (3)	23.1 (5)	0.388

Values are shown as median (interquartile range, IQR) or number (percent)

*BMI* Body mass index, *AD* Alzheimer’s disease, *NC* cognitively normal control

^a^ Mann-Whitney U test or *x*^2^ test

^b^ Missing data of *APOE* genotype (*n* = 1)

^c^ Missing data of *APOE* genotype (*n* = 2)

### Genera of GMB between AD and NC participants

After quality filtering and trimming, sequencing of the V3-V4 region of 16S rRNA gene generated 3.55 million sequence reads from 65 fecal samples with an average length of 419. At the 97% sequence similarity level, a total of 1014 OTUs were obtained and matched to 13 phyla, 20 classes, 35 orders, 71 families and 251 genera. The Venn diagram showed 53 unique OTUs in AD, 185 unique OTUs in NC and 776 shared OTUs between two groups (Additional file 1: Fig. S1A).

**Fig. 1:**
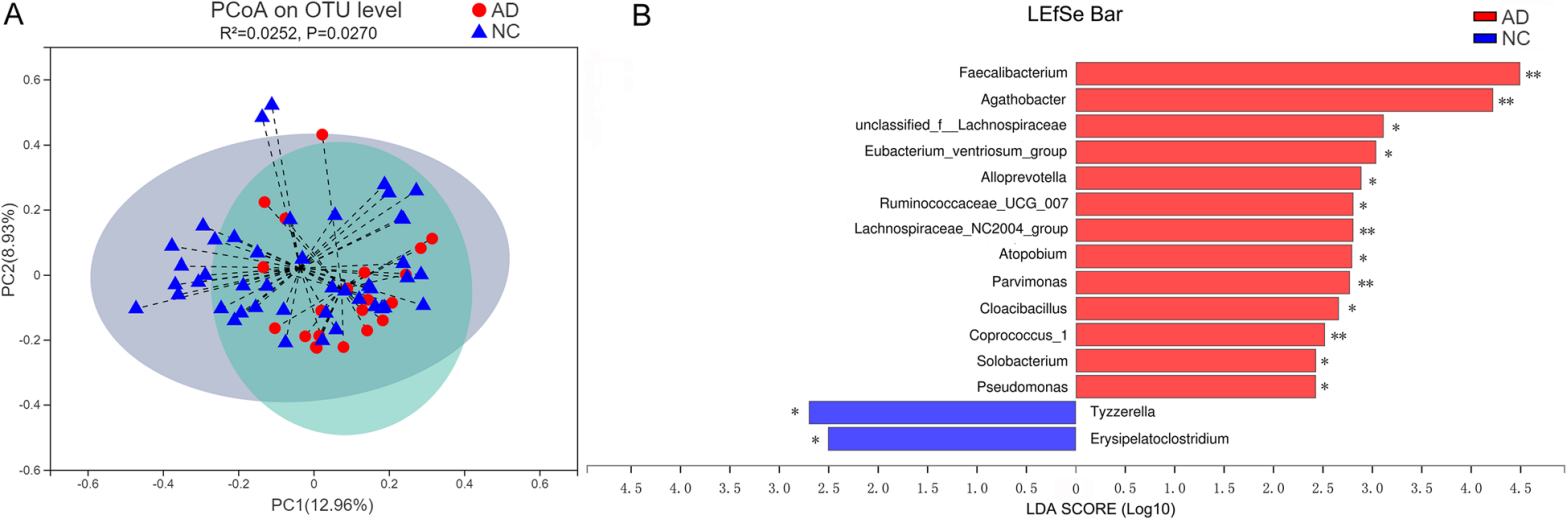
Gut microbial characteristics of Alzheimer’s disease (AD). **A** Principal co-ordinates (PCoA) analysis of microbiota in AD and NC participants (*n* = 21, AD; *n* = 44, NC, *P* = 0.027). **B** Taxonomic differences of gut microbiota bacterial between AD and NC participants analyzed by Linear discriminant analysis (LDA) effect size (LEfSe) (LDA > 2.5, *P* < 0.05). Histogram represents the LDA scores for differentially abundant genera. *P < 0.05; ** *P* < 0.01

The α-diversity did not differ between AD group and NC group (Additional file 2: Table S1). With respect to β-diversity, principal co-ordinates (PCoA) based on Bray-Curtis dissimilarity showed the significant differences of GMB composition between the fecal samples from AD participants and that from NC participants (PERMANOVA, *R*^*2*^ = 0.025, *P* = 0.027) (Fig. 1a). Meanwhile, control analyses were performed to determine whether the potentially confounding variables influenced this global microbial phenotype [28]. The results showed that the AD participants or NC participants were not clustered based on sex (Additional file 1: Fig. S1B), age (Additional file 1: Fig. S1C-D) or BMI (Additional file 1: Fig. S1E-F).

Furthermore, LEfSe analysis (Fig. 1b) showed that 15 genera significantly differed between AD group and NC group (LDA values > 2.5, Wilcoxon rank-sum test, *P* < 0.05; Additional file 3: Table S2). Specifically, AD participants exhibited 13 increased genera compared to NC participants, which belonged to bacterial families *Lachnospiraceae* (genera *Agathobacter, unclassified_f_Lachnospiraceae, Eubacterium_ventriosum_group, Lachnospiraceae_NC2004 and Coprococcus_1*), *Ruminococcaceae* (genera *Faecalibacterium and Ruminococcaceae_UCG-007), Prevotellaceae* (genus *Alloprevotella*), *Atopobiaceae* (genus *Atopobium*), *Clostridiales* (genus *Parvimonas*), *Synergistaceae* (genus *Cloacibacillus*), *Erysipelotrichaceae* (genus *Solobacterium*) and *Pseudomonadaceae* (genus *Pseudomonas*). In contrast, AD participants exhibited 2 decreased genera compared to NC participants, which belonged to bacterial families *Lachnospiraceae* (genus *Tyzzerella*) and *Erysipelotrichaceae* (genus *Erysipelatoclostridium*). At phyla level, these 15 differential bacterial genera were classified to *Firmicutes* (11/15, 73.3%), *Bacteroidetes* (1/15, 6.7%), *Actinobacteria* (1/15, 6.7%), *Proteobacteria* (1/15, 6.7%) and *Synergistetes* (1/15, 6.7%).

### Fecal Metabolome between AD and NC participants

**Fig. 2:**
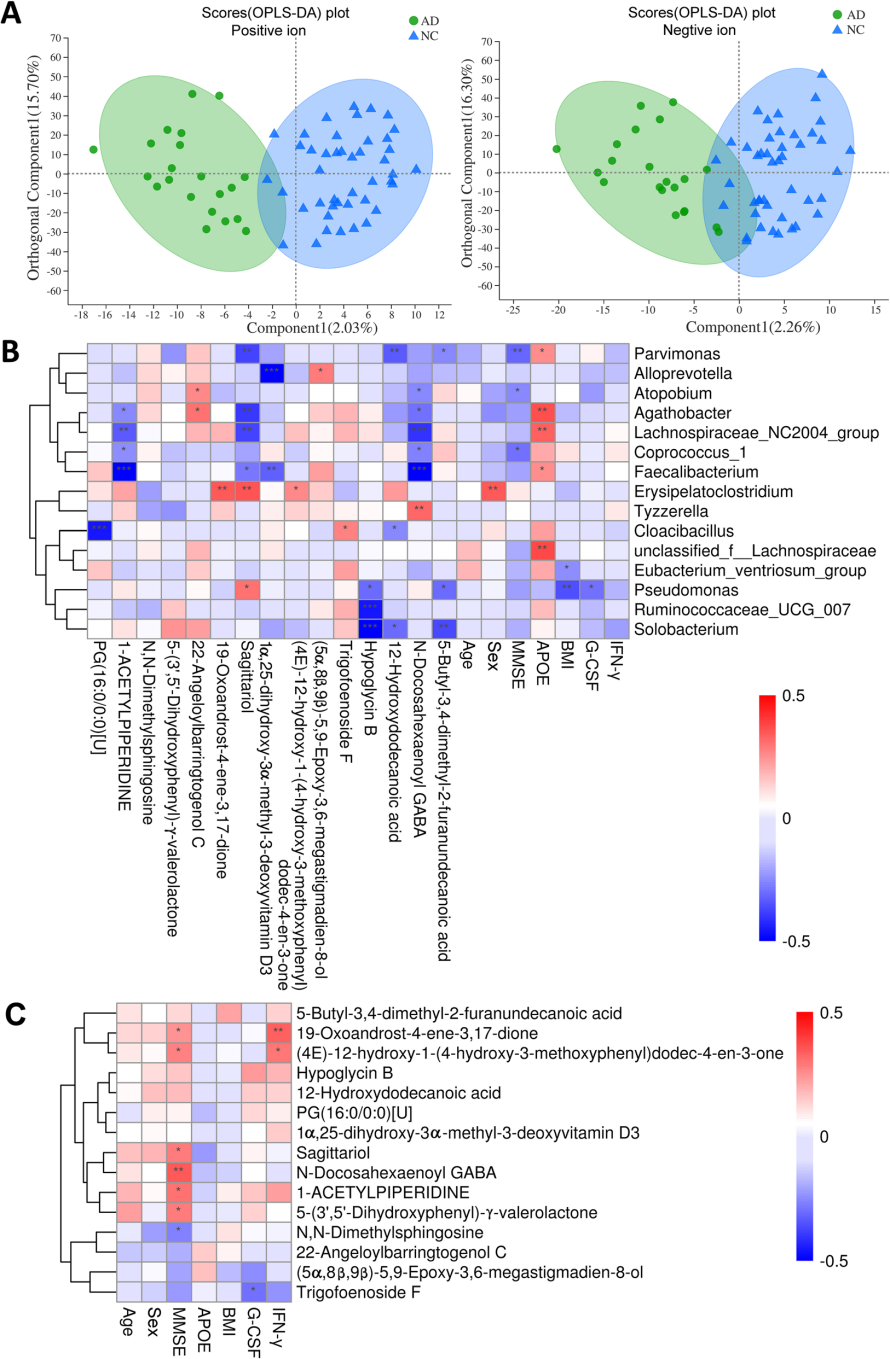
Characteristics of metabolites between AD participants and NC participants and the correlation analyses. **A** The orthogonal partial least-squares discriminant analysis (OPLS-DA) plot of fecal metabolites in comparisons of AD and NC groups (left: positive ion; right: negative ion). **B** Pearson correlation between 15 most differential genera and 15 differential metabolites, inflammatory cytokines, clinical characteristics linked with AD. The results were presented as a heatmap. Red squares indicate positive associations; blue squares indicate negative associations. *P < 0.05, ** *P* < 0.01, *** *P* < 0.001 denoted statistical significance. **C** Associations of fecal metabolites with inflammatory cytokines and clinical parameters. Red squares indicate positive associations; blue squares indicate negative associations. *P < 0.05; ** *P* < 0.01

Fecal metabolites, served as the metabolic output of both GMB and cellular metabolism occurring inside the human intestinal tract, is considered a functional readout of the GMB. As show in Fig. 2a, the OPLS-DA scores plot showed a clear variation between AD group and NC group (Permutation test: R^2^Y = 0.70, Q^2^Y = − 0.28 for positive ion, and R^2^Y = 0.74, Q^2^Y = − 0.15 for negative ion). Metabolome of AD participants showed 15 differential fecal metabolites relative to the Control group (*P* < 0.05, VIP > 1, Table 2), with decreased levels of 11 metabolites and increased level of 4 metabolites in AD. The implicated metabolites belonged to organic acids (3 metabolites: N-Docosahexaenoyl-GABA; Hypoglycin B and 12-Hydroxydodecanoic acid), lipids and lipid-like molecules (7 metabolites: 19-Oxoandrost-4-ene-3,17-dione; 5-Butyl-3,4-dimethyl-2-furanundecanoic acid; Trigofoenoside F; Sagittariol; 22-Angeloylbarringtogenol C; 1α,25-dihydroxy-3α-methyl-3-deoxyvitamin D3 and PG(16:0/0:0)[U]), Benzenoids (2 metabolites: (4E)-12-hydroxy-1-(4-hydroxy-3-methoxyphenyl)dodec-4-en-3-one and 5-3’, 5’-Dihydroxyphenyl-γ-valerolactone), Organic nitrogen (N,N-Dimethylsphingosine), Organoheterocyclic ((5α,8β,9β)-5,9-Epoxy-3,6-megastigmadien-8-ol) and Piperidine (1-ACETYLPIPERIDINE) (Table 2). KEGG pathway enrichment analysis revealed that the differentially expressed metabolites were involved in steroid hormone biosynthesis (19-Oxoandrost-4-ene-3,17-dione), N-Acyl amino acid metabolism (N-Docosahexaenoyl-GABA) and Piperidine metabolism (1-ACETYLPIPERIDINE) (Table 2).

**Table 2:** Alteration of Metabolites in AD subjects compared with cognitively normal controls

Compound	Metabolite	Predicted molecular pathways or biological functions^a^	VIP	FC (AD/NC)	*P*
**Lipids and lipid-like molecules**					
Androstane steroids	19-Oxoandrost-4-ene-3,17-dione	Steroid hormone biosynthesis	2.4	0.73	0.023
Fatty acids	5-Butyl-3,4-dimethyl-2-furanundecanoic acid	Antixoxidants and radical scavengers	2.1	0.80	0.034
Steroidal glycosides	Trigofoenoside F	A compound isolated from fenugreek seeds	1.5	1.07	0.011
Triterpenoids	22-Angeloylbarringtogenol C	A constituent of *C. sinensis*	3.3	1.22	0.042
Diterpenoids	Sagittariol	–	2.6	0.87	0.042
Vitamin D derivatives	1α,25-dihydroxy-3α-methyl-3-deoxyvitamin D3	–	2.7	0.87	0.031
–	PG(16:0/0:0)[U]	–	1.3	0.95	0.019
**Organic acids and derivatives**					
Amino acids	N-Docosahexaenoyl GABA	N-Acyl amino acid metabolism	2.3	0.81	0.001
Hydroxy acids	12-Hydroxydodecanoic acid	Substrate of glutathione-dependent formaldehyde dehydrogenase	1.6	0.85	0.047
Amino acids	Hypoglycin B	Natural toxins	1.4	0.86	0.037
**Benzenoids**					
Methoxyphenols	(4E)-12-hydroxy-1-(4-hydroxy-3-methoxyphenyl)dodec-4-en-3-one	A predicted metabolite generated by BioTransformer	2.1	0.67	0.020
Benzenediols	5-(3′,5′-Dihydroxyphenyl)-γ-valerolactone	A tea polyphenol metabolite detected in biological fluids	2.3	0.73	0.041
**Organic nitrogen compounds**					
Amines	N,N-Dimethylsphingosine	Regulation of sphingolipid-mediated functions	3.9	1.35	0.032
**Organoheterocyclic compounds**					
Benzopyrans	(5α,8β,9β)-5,9-Epoxy-3,6-megastigmadien-8-ol	–	1.8	1.23	0.045
**Piperidine**					
Piperidine	1-ACETYLPIPERIDINE	Piperidine metabolism	1.9	0.80	0.041

*AD* Alzheimer’s disease, *NC* cognitively normal control, *VIP* variable importance in the projection, *FC* fold change

^a^ Information referencing KEGG pathway database and HDBM database

### Relationship between altered gut microbial genera, fecal metabolites, clinical characteristics and inflammatory cytokines linked with AD

As shown in Fig. 2b, the Pearson correlation analyses indicated that 46.7% (7/15 genera) of altered bacterial genera were significantly associated with a series of altered fecal metabolites (*P* < 0.05, *r* > 0.30 or < − 0.30 [29], associated with more than one metabolite), suggesting a general correlation between gut bacterial file and metabolites. The coefficient of correlation and the *P* value between bacterial genera and fecal metabolites were presented in Additional file 4: Table S3 and Additional file 5: Table S4, respectively. These genera mainly belonged to *Lachnospiraceae* (*Lachnospiraceae_NC2004* and *Agathobacter*), *Erysipelotrichaceae* (*Erysipelatoclostridium* and *Solobacterium*), *Ruminococcaceae* (*Faecalibacterium*), *Pseudomonadaceae* (*Pseudomonas*) and *Clostridiales* (*Parvimonas*) at family level. Additionally, significant correlations were observed between some gut microbial genera and clinical parameters including sex, BMI, MMSE score, *APOE* genotype and G-CSF (Fig. 2b). These findings suggested that AD was simultaneously distinguished by changes in GMB and fecal metabolome.

As shown in Table 3, AD participants presented significantly decreased expression of serum G-CSF and IFN-γ compared to NC participants (*P* = 0.008 and *P* = 0.007, respectively) (Table 3). A significant negative correlation of Trigofoenoside F metabolite with G-CSF (*r* = − 0.301, *P* = 0.019) and a significant positive correlation of 19-Oxoandrost-4-ene-3,17-dione metabolite with IFN-γ (*r* = 0.339, *P* = 0.006) were observed (Fig. 2c, Additional file 6: Table S5 and Additional file 7: Table S6). These findings suggested that some fecal metabolites might affect systemic inflammatory reactions during AD development.

**Table 3:** Association of peripheral inflammatory cytokines and Alzheimer’s disease risk

Cytokines	Number of subjects	Mean		Median		*P value*^*a*^
	AD/NC	AD	NC	AD	NC	
IL-6	18/41	3.57	2.75	1.44	1.63	0.792
IL-8	18/43	7.38	6.77	6.09	5.99	0.794
IL-10	18/43	1.08	1.85	1.01	1.18	0.315
G-CSF	19/43	1.28	1.84	1.27	1.59	0.008
IL-7	19/43	2.14	2.22	2.05	2.05	0.667
IL-8	19/43	3.17	3.29	2.40	3.15	0.113
IL-12	19/42	1.32	1.50	0.95	0.95	0.849
IFN-γ	19/42	0.97	1.75	0.66	1.40	0.007
MCP-1	19/43	6.87	8.27	6.85	7.72	0.172
MIP-1β	19/43	8.33	10.95	6.45	8.28	0.194
TNF-α	19/39	2.27	2.67	1.61	2.12	0.314

*AD* Alzheimer’s disease, *NC* cognitively normal control

^a^ Comparison between AD group and NC group of median, using Mann-Whitney U test

### Potential contribution of fecal markers of AD

**Fig. 3:**
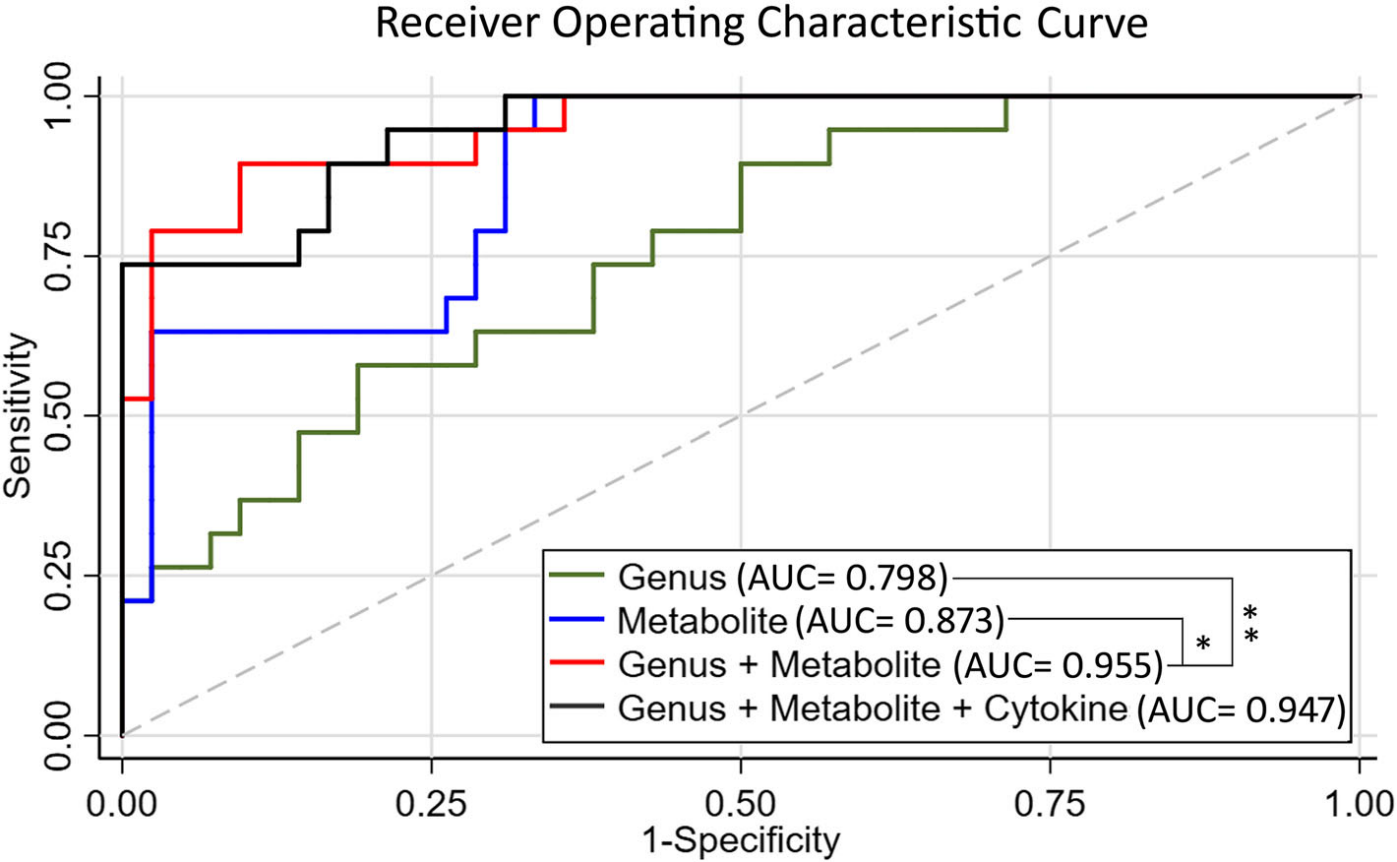
Diagnostic markers for AD. Receiver operating characteristic curves for genetic models among Chinese subjects in the testing set. Genus group represents 2 microbial (*Faecalibacterium* and *Pseudomonas).* Metabolite group represents 4 metabolites (N-Docosahexaenoyl GABA, 19-Oxoandrost-4-ene-3,17-dione, 22-Angeloylbarringtogenol C and Trigofoenoside F). Cytokine group represents G-CSF and IFN-γ

Among the newly implicated 17 fecal markers in our study, including of differential 7 fecal genera and their 8 significantly correlated fecal metabolites, and 2 fecal metabolites that were correlated with differential inflammatory cytokines in AD, combination of 6 fecal markers, including of 2 genera (*Faecalibacterium* and *Pseudomonas*) and 4 metabolites (N-Docosahexaenoyl GABA, 19-Oxoandrost-4-ene-3,17-dione, Trigofoenoside F and 22-Angeloylbarringtogenol C), was able to discriminate AD from NC with an AUC of 0.955 in these 65 subjects. The AUC of each separate microbial (AUC = 0.798) or metabolic markers (AUC = 0.873) was significantly lower than that of combination of microbial and metabolic markers, *P* < 0.001 and *P* = 0.033, respectively (Fig. 3). Addition of inflammatory cytokines did not significantly improve the discriminative power of the combined model (*P* = 0.857).

## Discussion

This study demonstrated that AD exhibited the disorder in microbial ecology, which may affect both metabolism and subsequent peripheral inflammatory cytokine in human subjects. Importantly, our study showed that the AUC for discriminating AD from NC was 0.955 for combination of fecal microbial markers and correlated metabolites (a total of six fecal markers), suggesting the potential of fecal markers as a non-invasive tool for screening or assistant diagnosis of AD. Further studies in large scale and longitudinal analysis can be justified to validate and improve the clinical utility of this fecal marker panel.

In our study, AD was characterized by increased 13 genera, of which 5 genera belonged to family *Lachnospiraceae*. Our results confirmed the previous report by chi et al. that the higher abundance of family *Lachnospiraceae* (general Fusicatenibacter, Blautia, and Dorea) was negatively associated with MMSE score [30]. In addition, a metagenomic study indicated the association of the family *Lachnospiraceae* with Type 2 diabetes and the intestinal colonization by the *Lachnospiraceae* contributes to the development of diabetes [31]. It is well known that diabetes and insulin resistance were risk factors of AD [32–34], therefore, a potential mechanism by which gut *Lachnospiraceae* influences AD development might be resulted from promoting the development of diabetes.

Our results showed that the abundance of genera *Pseudomonas* and *Faecalibacterium* increased in AD participants and these two genera contributed to fecal marker panel for effectively discrimination of AD from NC (Fig. 3). The genus *Pseudomonas* was found to be a possible risk factor of AD, as it increased both in fecal sample (our study results) and blood sample [12] from AD subjects, compared with that in cognitive normal controls. Functional studies reported that *P. aeruginosa* infection could increase endothelial tau phosphorylation and permeability [35, 36], which is a common pathophysiological mechanism in AD pathogenesis. In addition to the pathogenic *P. aeruginosa* strain, the genus *Pseudomonas* also comprises non-pahtogenic strains, such as *P. syringae strain* [37]. So far, little is known about how interactions between different pathogenic and non-pathogenic *Pseudomonas* strains within the host impact. Future studies should furtherly identify the specific pathogenic strain responsible to AD and explore the interaction between pathogenic and non-pathogenic strains on the occurrence of AD. With regard to *Faecalibacterium*, *F. prausnitzii (F. prausnitzii)* is the founding strain and has been studied extensively. *F. prausnitzii* plays a beneficial role in anti-inflammatory and promoting gut health [38, 39]. Several studies reported lower abundance of *F. prausnitzii* in patients with inflammatory bowel diseases [40, 41], type 2 diabetes [42] and colon cancer [43]. However, higher abundance in *F. prausnitzii* was reported in pediatric patients with Crohn’s Disease compared with controls [44, 45], indicating the detrimental role of *F. prausnitzii*, at least in some contexts. Lopez-Siles et al. [46] has found that patients with Crohn’s Disease may harbor a unique set of *F. prausnitzii* OTUs, as compared with controls. A study with deep metaproteomics approach has defined five available strain proteomes of *F. prausnitzii*. Therefore, inconsistent role of *F. prausnitzii* may be due to the different function of different strains of Faecalibacterium genera, which might be the potential confounders for the ROC curves evaluated in our study. Future research is needed to determine the specific strains that relevant to AD. Our results reported a higher abundance of gut *Faecalibacterium* in AD than NC group, providing additional evidence of detrimental role of *Faecalibacterium*. One explanation of may be that *Faecalibacterium* could reduce secretion of proinflammatory cytokines IFN-γ [38]. Then, reduced IFN-γ causes increase of Aβ deposits [47]. In fact, we also found the lower peripheral IFN-γ expression in AD compared with NC group, which supports this hypothetical explanation.

AD participants in our study showed alteration of fecal metabolome compared to NC participants. Correlation analysis provided a significant association of altered bacterial genera with altered metabolites. Furthermore, pathway analysis linked the alteration of metabolites to three metabolic pathways including Steroid hormone biosynthesis, N-Acyl amino acid metabolism and Piperidine metabolism. Together, these findings suggest the potential mechanism of GMB affecting occurrence or development of AD. Future functional studies are justified to formally verify that if and how these metabolic pathways participate in the occurrence or development of AD. Notably, among 15 altered metabolites in AD, 7 metabolites belong to lipid or lipid-like molecules, suggesting that lipid-mediated metabolic or synthetic processes in gut are linked with AD. The 19-Oxoandrost-4-ene-3, 17-dione, a decreased lipid-like molecular in AD compared with NC group in our study, was involved in estrogen biosynthesis. Estrogen is a potent factor that not only prevents vascular disease but also plays an important role in the preservation of neurons and reparation of neurons damaged by the disease [48]. A population-based study indicated higher frequency of estrogen use in non-patients compared with Alzheimer’s disease patients [49]. Thus, decreased 19-Oxoandrost-4-ene-3, 17-dione in gut may influence the AD pathology by downregulating the estrogen. 5-Butyl-3, 4-dimethyl-2-furanundecanoic acid, another decreased lipid-like metabolite in AD, is a furan fatty acid (F-acid). F-acids play an protective role for Alzheimer’s disease progression mainly by preventing lipid peroxidation [50, 51] and protecting polyunsaturated fatty acids (PUFAs) [52], suggesting possible mechanism for lower level of 5-Butyl-3, 4-dimethyl-2-furanundecanoic acid in AD than NC group. Regarding to amino acid metabolism, all three metabolites belonging to organic acids showed decreased level in AD, among which N-Docosahexaenoyl GABA was annotated to be involved in N-Acyl amino acid metabolism. Consistent with our results, N-acyl amino acids have anti-inflammatory and neuroprotective effects [53], but their biosynthesis and catabolism have not been fully elucidated [54].

Additionally, we observed decreased serum G-CSF and IFN-γ level in AD participants compared with NC participants. The consistent results were also observed in previous clinical and animal studies. Decreased plasma levels of G-CSF were previously reported in patients with early AD in comparison with healthy controls [55]. Moreover, in AD participants, G-CSF levels showed a significant inverse correlation with amyloid-β (Aβ1 − 42) levels in cerebrospinal fluid [55]. Mouse model experiment proved that subcutaneous administration of G-CSF into two different Aβ–induced AD mouse models could substantially rescue their cognitive/memory functions [56]. Additionally, in the stroke mouse model, administration of G-CSF could lead to neurogenesis near the injured area and the neurological and functional recovery [57–59]. With respect to IFN-γ, although the relationship between IFN-γ level and AD has not been reported, IFN-γ overexpression was found to reduce Aβ deposits and peripheral monocytes infiltration in amyloid precursor protein (APP) transgenic mice [47]. Additionally, in both 5XFAD and APP/PS1 mice model at intermediate stage of AD, reversion of decreased IFN-γ level by transient depletion of Treg cells could increase infiltration and recruitment of leukocyte to Aβ plaques and then alleviate the AD pathology [60]. The findings of these functional studies support our observation that IFN-γ is decreased in AD compared with NC group.

By integrating the correlation analyses of three groups of markers (GMB, fecal metabolites and peripheral inflammatory cytokines), we found two potential mechanistic pathways for the activity and function of GMB in AD pathogenesis. One pathway was that decrease of 19-Oxoandrost-4-ene-(3,17)-dione metabolite caused by low abundance of gut *Erysipelatoclostridium* might lead to reduction of peripheral IFN-γ level, which subsequently influenced AD pathogenesis. There are some evidences supporting the potential of this mechanism.19-Oxoandrost-4-ene-(3,17)-dione was involved in estrogen biosynthesis as we discussed above, besides, administration of estrogen to mouse could enhance the secretion of IFN-γ by mouse spleen cells [61] and by CD4+ human T cell clones [62]. The second pathway was that increase of Trigofoenoside F metabolite caused by high abundance of gut *Cloacibacillus* might lead to reduction of peripheral G-CSF level and then influenced AD pathogenesis. Though there are no other references supporting this mechanism for now, our findings have provided evidences for better understanding the role of microbiota in AD pathogenesis. It should be admitted that whether and how these mechanisms are involved in the occurrence or development of AD is still unclear, and further researches are needed.

There are several limitations to this study. First, it was a preliminary study with small sample size, and studies of larger population with different groups across Asia or with different ethnicity would give a better picture of the connection between GMB, metabolites and inflammatory cytokines, and also validate the predictive performance using fecal marker penal. Second, although our study suggests that the GMB and relative metabolic may be associated with the pathogenesis of AD, future intervention study and animal experiments are needed to identify whether this relationship connected by a causal relationship or is a simple incidental association. Third, functional study should be carried out to clarify the “bacterial-metabolite-cytokine” mechanism in AD pathogenesis and define the specific and effective targets for AD intervention and therapy. Fourth, the diagnosis of AD in this study was based on symptoms without disease severity information, and the neuroimaging markers should be included in further confirmatory studies for accurate diagnosis. Fifth, although we used LEfSe to identify the key bacterial taxa that altered between AD group and NC group, there are other methods to screen different bacterial taxa, such as PLS-DA VIP plot [63] or Random Forest analysis [64]. Future researches with follow-up data should be conducted to determine which method is better in determining key bacterial taxa responsible for AD risk. Finally, we performed the binary logistic regression analysis with the ROC curves to select and quantify which combination of fecal markers were best relevant to AD. Larger studies with cross-validation are justified to formally test the hypothesis and assess its predictive performance.

## Conclusions

In conclusion, our study provides preliminary evidence that AD exhibited changes in fecal microbial composition and its metabolic output, and these identified fecal markers enabled discriminating AD from NC with high accuracy. These findings improve our understanding of the role of GMB in AD pathogenesis, and provide a non-invasive way for early-stage clinical screening or assistant diagnosis of AD.

## Methods

### Study participants

From April 2018 to July 2019, 21 patients with AD from memory clinic at the Department of Neurology of Huashan Hospital and 44 cognitively normal controls (NC) from community participants with normal cognition from the Shanghai Aging Study [65] were recruited. These two groups did not differ in age, sex, and education years (*P* > 0.05, Table 1).

All participants recruited in study had similar diet habit. Exclusion criteria in this study included: use of antibiotics, probiotics, or prebiotics within a month before sampling; corticosteroid use, immune stimulating medications and immunosuppressive agents; use of antidepressant; gastrointestinal surgery in past 5 years; human immunodeficiency virus or serious bacterial infection in the medical history; inflammatory bowel disease, persistent, infectious gastroenteritis, colitis or gastritis; diarrhea or constipation at sampling.

The study protocol was approved by the Medical Ethics Committee of Huashan Hospital at Fudan University (IRB#2009-195) and the Ethics Committee of the Department of Public Health at Fudan University (IRB#2019-04-0739), Shanghai, China. All participants or participants’ legally acceptable representatives provided written informed consent to participate in this study. Subjects in this study were recruited based on the Shanghai Aging Study.

### Clinical interview and diagnosis of cognition

Neurologists conducted face-to-face interview for AD participants and controls to collect the clinical and demographic data. Neurologists and neuropsychologists reviewed the clinical and neuropsychological data and reached a consensus diagnosis of AD based on the DSM-IV and NINCDS-ADRDA criteria [66, 67]. The detailed information of clinical and neuropsychological assessment and diagnosis procedures were described elsewhere [68]. The genotypes of *APOE* were determined using the TaqMan SNP Genotyping assay [69]. *APOE* ε4 positive was defined as participants who have at least one ε4 allele.

### Sample collection

First morning fecal samples were collected in sterile fecal collection containers from each participant at home and packaged with frozen gel packs. Participants returned the fecal collection containers to Huashan hospital within 2 h. Upon receipt, samples were immediately subsampled into prepared sterile tube sand frozen at − 80 °C until further analysis. In the morning of the same day with fecal sample collection, the overnight fasting blood sample was collected from each participant by research nurses at Huashan Hospital. After centrifugation, serum were subsampled (300ul) into prepared sterile tubes and frozen at − 80 °C until analysis.

### DNA extraction and 16S rRNA gene sequencing

The E.Z.N.A.^@^ Stool DNA Kit (Omega Bio-Tek, Norcross, GA, USA) was used for microbial DNA extraction of fecal samples and extracted DNA samples were stored in elution buffer (Tris-HCL) provided in kit at − 80 °C until sequencing. The NanoDrop 2000 UV-vis spectrophotometer (Thermo Scientific, Wilmington, USA) was used for measuring DNA concentration. DNA integrity were shown and assessed by 1% agarose gel electrophoresis. The V3-V4 region of the 16S rRNA gene was amplified using the 6-bp barcoded universal primers: 338 forward (5′-ACTCCTACGGGAGGCAGCAG-3′) and 806 reverse (5′-GGACTACHVGGGTWTCTAAT-3′). PCR reaction was carried out in a 20 μL reaction volumes containing 5 × FastPfu Buffer (4 μL), 2.5 mM dNTPs (2 μL), each of 5 μM primer (0.8 μL), FastPfu Polymerase (0.4 μL) and 10 ng of template DNA. The PCR amplicons were then purified by the AxyPrep DNA Gel Extraction Kit (Axygen Biosciences, USA) and quantified by the QuantiFluor™-ST fluorometer (Promega Corporation, USA). The final equimolar pool was sequenced (paired end, 2 × 300-bp) using Illumina Miseq sequencing technology (Illumina, San Diego, USA).

### Bioinformatics processing and analysis of 16S rRNA gene sequence

Raw FastQ files were demultiplexed, quality-filtered by Trimmomatic, and merged using FLASH. The following standards were applied in data processing: 1) Reads were trimmed with an quality score lower than 20 over a 50-bp sliding window; 2) The primers were completely matched, but 2 mismatched nucleotides were allowed [70], and sequence reads with ambiguous bases were excluded from downstream analysis; 3) Sequences containing overlaps of > 10 bp were merged in accordance with their overlaps. Finally, remaining sequences were clustered into operational taxonomic units (OTUs) at 97% similarity level using UPARSE (version 7.1, http://drive5.com/uparse/). The Taxonomies of each 16S rRNA gene sequence was analyzed using the RDP Classifier Algorithm (http://rdp.cme.msu.edu/) at a confidence threshold of 70% [71] against the Silva (SSU132) 16S rRNA database (Release132 http://www.arb-silva.de).

OTUs counts were normalized before testing group-significant taxa and rarified to an equal number of 28,277 sequencing reads per each sample before calculating diversity indices. Sequence data were analyzed using the Quantitative Insights Into Microbial Ecology platform (QIIME; V1.9.1) [72] and R packages (v3.2.0), and the free online Majorbio I-Sanger Cloud Platform (www.i-sanger.com). α-diversity were measured using the Ace and Chao indices for richness and using the Shannon and Invsimpson for diversity by Wilcoxon test on the rarefied OTU data. β-diversity (between-habitat diversity) shows the shared diversity between bacterial population in terms of ecological distance. β-diversity were calculated by Bray-Curtis dissimilarity algorithms on the rarefied OTU data, and reported according to PCoA with 95% confidence interval (CI) ellipses. Statistical differences in β-diversity metrics between AD group and NC group were detected using permutational multivariate analysis of variance (PERMANOVA) [73] by the R “vegan” package. Statistically significant differences in specific bacterial taxa between AD group and NC group were analyzed using linear discriminant analysis (LDA) effect size (LEfSe) [74]. LDA scores were calculated by LDA effect size. Difference with LDA values > 2.5 at *P* value < 0.05 were considered significant.

### Metabolomics profiling based on UPLC-MS

Fecal samples (50 mg) were added with 400 μL methanol-water (4:1, v/v) and the supernatant was obtained according to the following procedure: 6 min for homogenization, 30 min for ultrasonically extraction on ice, 30 min for keeping at − 20 °C and 15 min for centrifugation at 13000 rpm at 4 °C. Next, 200 μL supernatant was used for UPLC-MS analysis and a mixture of all extraction aliquots was used as a quality control (QC) sample. LC-MS-based fecal metabolic profiling were performed on an Ethylene Bridged Hybrid C18 column (100 mm × 2.1 mm .id., 1.7 μm internal diameter, Waters Corp., Milford, USA), coupled to a Triple TOF™ 5600 mass spectrometer system (AB SICEX, USA). The column temperature was 40 °C. Injection volume of the prepared sample was 20 μL. The mobile phase included solvent A (water containing 0.1% (v/v) formic acid) and solvent B (acetonitrile/isopropanol (1:1) containing 0.1% (v/v) formic acid) and the flow rate was 0.40 mL/min. The elution gradient was 5% B–20% B from 0 to 3 min, 20% B–95% B from 3 to 9 min, holding at 95% B till 13.0 min, then decreased to 5% B in 0.1 min, and holding at 5% B over13.1–16 min. Both positive and negative ion scanning modes were implemented for MS signal acquisition. To obtain information regarding system repeatability, QC samples were injected at every 8 analytical samples throughout the analytical run.

### Metabolomics data analysis and metabolite identification

The raw MS data were processed by Progenesis QI data analysis software (Waters Corp, Milford, MA, USA). Data matrix including peak intensity, retention time of compounds and mass-to-charge ratio were obtained. The detail of data matrix processing procedure was described in our previous published paper [75]. The Data matrix were log10 transformed for subsequent analysis. Data analysis were performed at Majorbio I-Sanger Cloud platform (www.i-sanger.com). The orthogonal partial least-squares discriminant analysis (OPLS-DA) was applied to detect variation between AD group and NC group. Although the significant FDR-adjusted *P* value for metabolites between two groups were not observed (data not shown), we combined univariate methods that analyze each metabolite separately and multivariate OPLS-DA methods to reduce false positive results and improve the reliability of the results [76]. Based on the OPLS-DA analysis, metabolites with variable importance in the projection (VIP) > 1.0, *P* values of < 0.05 (Student’s t-test) referring to published paper [77] were considered to be significantly differed between the AD and NC groups. Fold-change (FC) of metabolite in AD group compared with NC group were analyzed using the data before log10 transformation. Metabolites were identified using the METLIN online database (https://metlin.scripps.edu/) and HDBM database. The molecular pathways and biological functions of differential metabolites were predicted by KEGG pathway database and HDBM database.

### Cytokine assay

IL-6, IL-8, IL-10, and G-CSF were detected using the CBA Human Cytokine Kit (BD Biosciences, San Jose, CA) and IL-7, IL-8, IL- 12p70, IFN-γ, MCP-1, MIP-1β, TNF-α were detected using Bio-Plex Pro Human Cytokine kit (Bio-Rad, Hercules, California). CBA kit assay was performed on BD Accuri C6 flow cytometer (BD Biosciences) according to the manufacturer’s instructions and the data analysis was done by using BD CBA Software to generate graphical and tabular formats. The set of calibrators was applied to create the standard curves, and the results were obtained from test samples. For Bio-Plex kit assay, serum samples were diluted 1:1 for measurement, and 50 μL of the diluted samples was added to each well. Data were obtained by a Bio-Plex 200 system (Bio-Rad) and analyzed with the Bio-Plex Manager Software Version 5.0.

### Statistical analysis

For comparisons of demographic and clinical data between groups, continuous variables are described with median or interquartile range (IQR), and compared between groups using Mann-Whitney U test. Categorical variables were analyzed by Chi-square test. Serum concentrations of cytokines were compared between AD group and NC group, using the Mann–Whitney U test. All these statistical analyses were performed using SPSS version 21.0 (SPSS, Chicago, IL, US). A two-sided *P*-value of < 0.05 was considered to be statistically significant. For correlation analysis, Pearson correlation test using the R package was performed to calculate the exact correlation coefficient and the corresponding *P* value (set to 0.05) and the Heatmap plots were generated using the R packages. A binary logistic regression with backward selection was performed to determine which combination of fecal markers were best relevant to predict AD. Receiver operating characteristic (ROC) analyses were performed using STATA version 15.0. The sensitivity, specificity and the area under the ROC curve (AUC) were calculated to evaluate the usefulness of the model for identification of the presence of AD.

## Supplementary Information

**Additional file 1: Fig. S1.** The OUTs distribution and the control analyses of global microbial phenotypes.

**Additional file 2: Table S1.** α-phylogenetic diversity analysis.

**Additional file 3: Table S2.** Linear discriminant analysis effect size (LEfSe) analysis for differential bacteria between AD group and NC group.

**Additional file 4: Table S3.** The coefficient of correlation between microbial genus and fecal metabolites, clinical parameters and inflammatory cytokines.

**Additional file 5: Table S4.** The *P* valued of correlation between microbial genus and fecal metabolites, clinical parameters and inflammatory cytokines.

**Additional file 6: Table S5.** The coefficient of correlation between fecal metabolites and clinical parameters and inflammatory cytokines.

**Additional file 7: Table S6.** The *P* valued of correlation between fecal metabolites, clinical parameters and inflammatory cytokines.

## Data Availability

The raw data of 16S rRNA gene sequence are available from the Sequence Read Archive (SRA) database of National Center for Biotechnology Information (NCBI) official website (accession number: SRP252374).

## Abbreviations

AD: Alzheimer’s disease
NC: Cognitively normal control
Aβ: Amyloid-β
GMB: Gut microbiota
MCI: Mild cognitive impairment
rRNA: Ribosomal RNA
BBB: Blood-brain barrier
PCR: Polymerase Chain Reaction
SRA: Sequence Read Archive
OTUs: Operational taxonomic units
PCoA: Principal co-ordinates analysis
PERMANOVA: Permutational multivariate analysis of variance
LEfSe: Linear discriminant analysis effect size
QC: Quality control
OPLS-DA: Orthogonal partial least-squares discriminant analysis
SD: Standard deviation
ROC: Receiver operating characteristic
AUC: Area under the receiver operating characteristic curve
PUFAs: Protecting polyunsaturated fatty acids
APP: Amyloid precursor protein
