# Supplementary material for: Disturbed microbial ecology in Alzheimer’s disease: evidence from the gut microbiota and fecal metabolome
Source: BMC Microbiol. 2021 Aug 12;21:226. doi: 10.1186/s12866-021-02286-z (PMC8361629; doi:10.1186/s12866-021-02286-z)
Supplement: Supplementary file 1 — Additional file 1: Fig. S1. The OUTs distribution and the control analyses of global microbial phenotypes. [file 12866_2021_2286_MOESM1_ESM.docx]

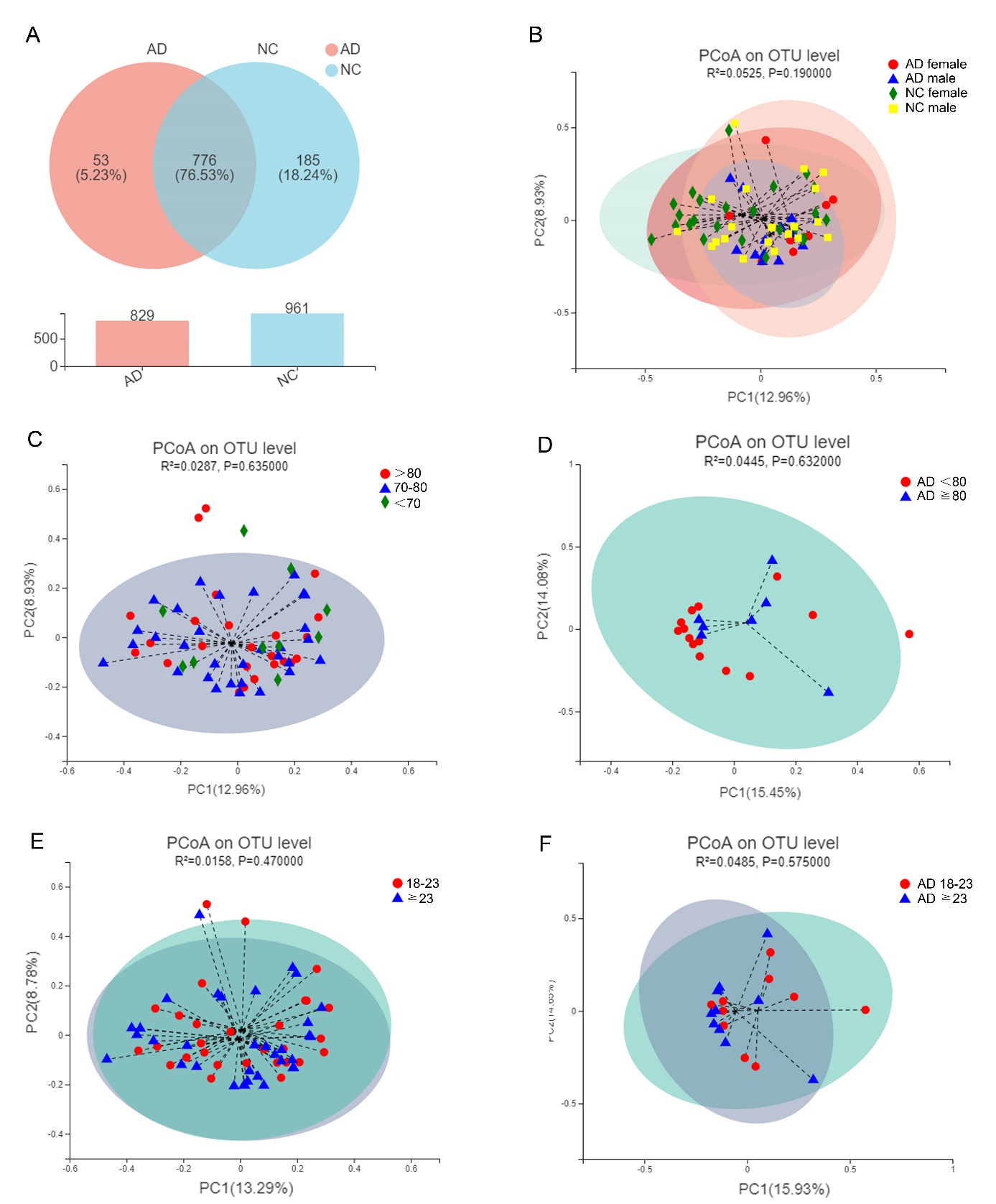


**Fig. S1.** The OUTs distribution and the control analyses of overall microbial phenotypes by PCoA with 95% confidence interval (CI) ellipses based on Bray-Curtis dissimilarity. **(A)** Venn diagram showing the unique and shared OTUs between AD participants and NC participants. **(B)** The overall microbial phenotypes based on sex (n=13, AD Male; n=8, AD Female; n=20, NC Male; n=24, NC Female). **(C-D)** The overall microbial phenotypes of all subjects (C) or AD group (D) based on age (All subjects: n=10, <70; n=31, 70-80; n=24, >80. AD group: n=14, <80; n=7,≧80). **(E-F)** The overall microbial phenotypes of all subjects (E) or AD group (F) based on Body Mass Index (BMI) (All subjects: n=29, BMI:18-23; n=34, BMI≧23; n=2, missing data. AD group: n=10, BMI:18-23; n=10, BMI≧23; n=1 missing data).
