# Supplementary material for: Disturbed microbial ecology in Alzheimer’s disease: evidence from the gut microbiota and fecal metabolome
Source: BMC Microbiol. 2021 Aug 12;21:226. doi: 10.1186/s12866-021-02286-z (PMC8361629; doi:10.1186/s12866-021-02286-z)
Supplement: Supplementary file 2 — Additional file 2: Table S1. α-phylogenetic diversity analysis. [file 12866_2021_2286_MOESM2_ESM.docx]

**Table S1** α-phylogenetic diversity analysis.

|  | **AD** |  | **NC** |  |
| --- | --- | --- | --- | --- |
| **Estimators** | **Mean (SD)** |  | **Mean (SD)** | ***P* value** |
| Shannon | 3.51 (0.51) |  | 3.35 (0.64) | 0.453 |
| Ace | 294.72 (85.26) |  | 277.73 (95.34) | 0.496 |
| Hao | 293.28 (84.42) |  | 281.96 (101.5) | 0.659 |
| Invsimpson | 17.15 (7.89) |  | 16.021 (9.00) | 0.580 |

*AD, Alzheimer’s disease; NC, cognitively normal control.*
