# Supplementary material for: Disturbed microbial ecology in Alzheimer’s disease: evidence from the gut microbiota and fecal metabolome
Source: BMC Microbiol. 2021 Aug 12;21:226. doi: 10.1186/s12866-021-02286-z (PMC8361629; doi:10.1186/s12866-021-02286-z)
Supplement: Supplementary file 3 — Additional file 3: Table S2. Linear discriminant analysis effect size (LEfSe) analysis for differential bacteria between AD group and NC group. [file 12866_2021_2286_MOESM3_ESM.docx]

**Table S2** Linear discriminant analysis effect size (LEfSe) analysis for differential bacteria between AD and NC groups.

| **Genera bacterial name** | **Group** | **Mean** | **LDA value** | ***P* value** |
| --- | --- | --- | --- | --- |
| Lachnospiraceae_NC2004_group | AD | 3.36 | 2.807 | 0.003 |
| Faecalibacterium | AD | 5.07 | 4.494 | 0.006 |
| Agathobacter | AD | 4.78 | 4.222 | 0.007 |
| Coprococcus_1 | AD | 3.00 | 2.520 | 0.009 |
| Parvimonas | AD | 0.83 | 2.773 | 0.011 |
| Erysipelatoclostridium | NC | 2.93 | 2.507 | 0.030 |
| Pseudomonas | AD | 2.22 | 2.429 | 0.030 |
| Eubacterium__ventriosum_group | AD | 3.65 | 3.037 | 0.032 |
| Solobacterium | AD | 1.27 | 2.429 | 0.032 |
| Alloprevotella | AD | 3.03 | 2.889 | 0.040 |
| Ruminococcaceae_UCG_007 | AD | 1.18 | 2.808 | 0.039 |
| Unclassified_f__Lachnospiraceae | AD | 3.94 | 3.114 | 0.043 |
| Tyzzerella | NC | 3.10 | 2.701 | 0.044 |
| Atopobium | AD | 1.07 | 2.795 | 0.050 |
| Cloacibacillus | AD | 3.13 | 2.661 | 0.049 |

*AD, Alzheimer’s disease; NC, cognitively normal control; LDA, linear discriminant analysis.*
