# Supplementary material for: Disturbed microbial ecology in Alzheimer’s disease: evidence from the gut microbiota and fecal metabolome
Source: BMC Microbiol. 2021 Aug 12;21:226. doi: 10.1186/s12866-021-02286-z (PMC8361629; doi:10.1186/s12866-021-02286-z)
Supplement: Supplementary file 4 — Additional file 4: Table S3. The coefficient of correlation between microbial genus and fecal metabolites, clinical parameters and inflammatory cytokines. [file 12866_2021_2286_MOESM4_ESM.docx]

**Table S3** The coefficient of correlation between microbial genus and fecal metabolites, clinical parameters and inflammatory cytokines.

|  | **PG(16:0/0:0)[U]** | **1-ACETYLPIPERIDINE** | **N,N-Dimethylsphingosine** | **5-(3',5'-Dihydroxyphenyl)- γ-valerolactone** | **22-Angeloylbarringtogenol C** | **19-Oxoandrost-4-ene-3,17-dione** | **Sagittariol** | **1α,25-dihydroxy-3α-methyl-3-deoxyvitamin D3** | **(4E)-12-hydroxy-1-(4-hydroxy-3-methoxyphenyl)dodec-4-en-3-one** | **(5α,8β,9β)-5,9-Epoxy-3,6-megastigmadien-8-ol** | **Trigofoenoside F** |
| --- | --- | --- | --- | --- | --- | --- | --- | --- | --- | --- | --- |
| Parvimonas | -0.105 | -0.089 | 0.101 | -0.231 | 0.150 | -0.051 | -0.375* | -0.209 | -0.006 | -0.060 | -0.036 |
| Alloprevotella | -0.002 | -0.150 | 0.128 | 0.076 | 0.147 | -0.052 | -0.127 | -0.494* | -0.153 | 0.284 | -0.020 |
| Atopobium | -0.035 | -0.017 | 0.141 | 0.000 | 0.268 | -0.165 | -0.129 | -0.100 | -0.066 | 0.053 | 0.043 |
| Agathobacter | -0.057 | -0.254 | 0.129 | 0.052 | 0.307* | 0.057 | -0.396* | -0.146 | 0.042 | 0.144 | 0.180 |
| Lachnospiraceae_NC2004_group | 0.060 | -0.337* | 0.030 | 0.045 | 0.187 | 0.180 | -0.375* | -0.115 | 0.161 | 0.079 | 0.187 |
| Coprococcus_1 | 0.040 | -0.244 | 0.072 | -0.152 | -0.125 | -0.136 | -0.202 | 0.096 | -0.133 | 0.069 | 0.013 |
| Faecalibacterium | 0.152 | -0.507* | 0.053 | -0.128 | 0.003 | 0.033 | -0.287 | -0.329* | -0.095 | 0.228 | -0.120 |
| Erysipelatoclostridium | 0.105 | 0.216 | -0.212 | -0.045 | -0.004 | 0.349* | 0.353* | 0.068 | 0.269 | 0.147 | -0.165 |
| Tyzzerella | -0.027 | 0.135 | -0.200 | -0.239 | -0.091 | 0.148 | -0.006 | 0.081 | 0.135 | 0.107 | 0.077 |
| Cloacibacillus | -0.458* | 0.018 | 0.062 | -0.093 | -0.047 | -0.087 | -0.039 | 0.089 | 0.054 | -0.113 | 0.270 |
| unclassified_f__Lachnospiraceae | 0.022 | -0.058 | 0.081 | 0.002 | 0.188 | 0.017 | 0.002 | 0.091 | -0.040 | -0.052 | -0.052 |
| Eubacterium_ventriosum_group | 0.152 | -0.133 | -0.067 | -0.113 | 0.147 | 0.018 | -0.128 | 0.007 | -0.046 | 0.019 | 0.229 |
| Pseudomonas | -0.051 | 0.092 | 0.018 | -0.052 | 0.069 | -0.124 | 0.302* | 0.016 | -0.071 | -0.075 | 0.075 |
| Ruminococcaceae_UCG_007 | 0.020 | -0.144 | -0.174 | 0.154 | -0.052 | -0.069 | -0.125 | -0.053 | -0.054 | 0.094 | 0.193 |
| Solobacterium | 0.042 | 0.105 | 0.036 | 0.240 | 0.224 | -0.098 | 0.024 | -0.201 | -0.166 | -0.050 | 0.151 |
| continue |  |  |  |  |  |  |  |  |  |  |  |
|  | Hypoglycin B | 12-Hydroxydodecanoic acid | N-Docosahexaenoyl GABA | 5-Butyl-3,4-dimethyl-2-furanundecanoic acid | Age | Sex | MMSE | APOE | BMI | G-CSF | IFN-g |
| Parvimonas | -0.119 | -0.337 | -0.210 | -0.255 | -0.196 | -0.100 | -0.319* | 0.255 | 0.008 | 0.071 | -0.164 |
| Alloprevotella | -0.065 | -0.130 | -0.201 | -0.162 | -0.151 | 0.009 | -0.125 | -0.098 | -0.013 | -0.162 | -0.159 |
| Atopobium | -0.071 | -0.163 | -0.255 | 0.127 | 0.064 | -0.208 | -0.259 | 0.000 | 0.056 | -0.230 | -0.102 |
| Agathobacter | 0.087 | -0.229 | -0.291 | -0.026 | -0.060 | -0.242 | -0.210 | 0.368* | -0.161 | -0.112 | -0.016 |
| Lachnospiraceae_NC2004_group | 0.106 | -0.174 | -0.402* | 0.105 | -0.044 | -0.111 | -0.163 | 0.337* | -0.068 | -0.087 | 0.067 |
| Coprococcus_1 | -0.022 | 0.085 | -0.264 | 0.151 | -0.084 | -0.173 | -0.293 | 0.205 | 0.101 | 0.005 | 0.096 |
| Faecalibacterium | 0.057 | -0.052 | -0.525* | 0.076 | -0.068 | -0.160 | -0.202 | 0.255 | -0.082 | -0.061 | 0.055 |
| Erysipelatoclostridium | 0.015 | 0.233 | 0.006 | -0.045 | 0.052 | 0.345* | 0.097 | 0.015 | -0.162 | 0.011 | -0.006 |
| Tyzzerella | 0.067 | 0.043 | 0.323* | -0.029 | -0.083 | -0.069 | 0.059 | -0.017 | -0.060 | -0.053 | 0.097 |
| Cloacibacillus | -0.087 | -0.254 | 0.015 | -0.084 | -0.001 | 0.108 | -0.088 | 0.226 | -0.097 | -0.057 | -0.121 |
| unclassified_f__Lachnospiraceae | 0.033 | 0.044 | 0.037 | 0.053 | 0.177 | 0.038 | -0.196 | 0.376* | -0.086 | 0.050 | 0.006 |
| Eubacterium_ventriosum_group | 0.025 | -0.081 | -0.184 | -0.092 | 0.187 | 0.030 | -0.174 | 0.208 | -0.250 | -0.072 | -0.077 |
| Pseudomonas | -0.302 | -0.085 | 0.081 | -0.307* | -0.023 | 0.026 | -0.212 | -0.167 | -0.352* | -0.303* | -0.179 |
| Ruminococcaceae_UCG_007 | -0.452* | -0.128 | -0.069 | -0.023 | 0.009 | -0.054 | -0.112 | 0.175 | -0.014 | -0.164 | -0.073 |
| Solobacterium | -0.495* | -0.306* | -0.026 | -0.367* | -0.095 | -0.041 | -0.189 | 0.073 | 0.018 | -0.166 | -0.214 |

* coefficient of correlation > 0.30 or < -0.30.
