# Supplementary material for: Disturbed microbial ecology in Alzheimer’s disease: evidence from the gut microbiota and fecal metabolome
Source: BMC Microbiol. 2021 Aug 12;21:226. doi: 10.1186/s12866-021-02286-z (PMC8361629; doi:10.1186/s12866-021-02286-z)
Supplement: Supplementary file 5 — Additional file 5: Table S4. The P valued of correlation between microbial genus and fecal metabolites, clinical parameters and inflammatory cytokines. [file 12866_2021_2286_MOESM5_ESM.docx]

**Table S4** The *P* valued of correlation between microbial genus and fecal metabolites, clinical parameters and inflammatory cytokines.

|  | **PG(16:0/0:0)[U]** | **1-ACETYLPIPERIDINE** | **N,N-Dimethylsphingosine** | **5-(3',5'-Dihydroxyphenyl)- γ-valerolactone** | **22-Angeloylbarringtogenol C** | **19-Oxoandrost-4-ene-3,17-dione** | **Sagittariol** | **1α,25-dihydroxy-3α-methyl-3-deoxyvitamin D3** | **(4E)-12-hydroxy-1-(4-hydroxy-3-methoxyphenyl)dodec-4-en-3-one** | **(5α,8β,9β)-5,9-Epoxy-3,6-megastigmadien-8-ol** | **Trigofoenoside F** |
| --- | --- | --- | --- | --- | --- | --- | --- | --- | --- | --- | --- |
| Parvimonas | 0.406 | 0.480 | 0.424 | 0.064 | 0.232 | 0.689 | 0.002^**^ | 0.095 | 0.963 | 0.635 | 0.776 |
| Alloprevotella | 0.985 | 0.232 | 0.310 | 0.548 | 0.244 | 0.681 | 0.312 | < 0.001^***^ | 0.224 | 0.022^*^ | 0.874 |
| Atopobium | 0.781 | 0.896 | 0.262 | 0.997 | 0.031^*^ | 0.189 | 0.305 | 0.427 | 0.602 | 0.672 | 0.736 |
| Agathobacter | 0.651 | 0.041^*^ | 0.304 | 0.679 | 0.013^*^ | 0.654 | 0.001^**^ | 0.245 | 0.741 | 0.251 | 0.151 |
| Lachnospiraceae_NC2004_group | 0.636 | 0.006^**^ | 0.810 | 0.721 | 0.137 | 0.151 | 0.002^**^ | 0.360 | 0.2.00 | 0.533 | 0.135 |
| Coprococcus_1 | 0.753 | 0.050^*^ | 0.569 | 0.227 | 0.319 | 0.281 | 0.107 | 0.448 | 0.291 | 0.583 | 0.919 |
| Faecalibacterium | 0.226 | < 0.001^***^ | 0.677 | 0.311 | 0.979 | 0.794 | 0.020^*^ | 0.007^**^ | 0.454 | 0.068 | 0.342 |
| Erysipelatoclostridium | 0.406 | 0.084 | 0.090 | 0.720 | 0.977 | 0.004^**^ | 0.004^**^ | 0.591 | 0.030^*^ | 0.244 | 0.190 |
| Tyzzerella | 0.832 | 0.283 | 0.110 | 0.055 | 0.470 | 0.241 | 0.96 | 0.519 | 0.283 | 0.396 | 0.541 |
| Cloacibacillus | < 0.001^***^ | 0.886 | 0.621 | 0.462 | 0.709 | 0.492 | 0.757 | 0.478 | 0.667 | 0.372 | 0.030^*^ |
| unclassified_f__Lachnospiraceae | 0.864 | 0.649 | 0.521 | 0.986 | 0.134 | 0.893 | 0.988 | 0.470 | 0.751 | 0.680 | 0.680 |
| Eubacterium_ventriosum_group | 0.226 | 0.289 | 0.598 | 0.369 | 0.241 | 0.887 | 0.310 | 0.954 | 0.715 | 0.881 | 0.067 |
| Pseudomonas | 0.688 | 0.466 | 0.888 | 0.682 | 0.585 | 0.325 | 0.015^*^ | 0.901 | 0.574 | 0.554 | 0.554 |
| Ruminococcaceae_UCG_007 | 0.874 | 0.253 | 0.166 | 0.221 | 0.682 | 0.585 | 0.322 | 0.677 | 0.671 | 0.458 | 0.123 |
| Solobacterium | 0.738 | 0.404 | 0.778 | 0.054 | 0.073 | 0.44 | 0.851 | 0.108 | 0.186 | 0.692 | 0.23 |
| continue | | | | | | | | | | | |
|  | Hypoglycin B | 12-Hydroxydodecanoic acid | N-Docosahexaenoyl GABA | 5-Butyl-3,4-dimethyl-2-furanundecanoic acid | Age | Sex | MMSE | APOE | BMI | G-CSF | IFN-g |
| Parvimonas | 0.347 | 0.006^**^ | 0.093 | 0.041^*^ | 0.117 | 0.427 | 0.010^**^ | 0.040^*^ | 0.951 | 0.572 | 0.193 |
| Alloprevotella | 0.606 | 0.303 | 0.109 | 0.199 | 0.231 | 0.944 | 0.321 | 0.440 | 0.918 | 0.197 | 0.205 |
| Atopobium | 0.574 | 0.194 | 0.040^*^ | 0.312 | 0.610 | 0.096 | 0.038^*^ | 1.000 | 0.660 | 0.065 | 0.420 |
| Agathobacter | 0.490 | 0.066 | 0.019^*^ | 0.839 | 0.635 | 0.052 | 0.093 | 0.003^**^ | 0.199 | 0.375 | 0.900 |
| Lachnospiraceae_NC2004_group | 0.399 | 0.166 | 0.001^***^ | 0.405 | 0.730 | 0.378 | 0.194 | 0.006^**^ | 0.588 | 0.489 | 0.598 |
| Coprococcus_1 | 0.864 | 0.499 | 0.034^*^ | 0.229 | 0.504 | 0.169 | 0.018^*^ | 0.102 | 0.422 | 0.967 | 0.448 |
| Faecalibacterium | 0.649 | 0.682 | < 0.001^***^ | 0.547 | 0.589 | 0.204 | 0.107 | 0.041^*^ | 0.518 | 0.632 | 0.661 |
| Erysipelatoclostridium | 0.908 | 0.062 | 0.961 | 0.720 | 0.683 | 0.005^**^ | 0.441 | 0.907 | 0.197 | 0.931 | 0.965 |
| Tyzzerella | 0.595 | 0.733 | 0.009^**^ | 0.817 | 0.511 | 0.584 | 0.643 | 0.890 | 0.636 | 0.677 | 0.440 |
| Cloacibacillus | 0.493 | 0.041^*^ | 0.906 | 0.507 | 0.994 | 0.39 | 0.484 | 0.071 | 0.440 | 0.65 | 0.336 |
| unclassified_f__Lachnospiraceae | 0.792 | 0.727 | 0.768 | 0.677 | 0.158 | 0.761 | 0.117 | 0.002^**^ | 0.494 | 0.691 | 0.961 |
| Eubacterium_ventriosum_group | 0.846 | 0.519 | 0.143 | 0.464 | 0.136 | 0.813 | 0.165 | 0.096 | 0.045^*^ | 0.566 | 0.542 |
| Pseudomonas | 0.014^*^ | 0.499 | 0.523 | 0.013^*^ | 0.855 | 0.839 | 0.091 | 0.183 | 0.004^**^ | 0.016^*^ | 0.153 |
| Ruminococcaceae_UCG_007 | < 0.001^***^ | 0.311 | 0.584 | 0.856 | 0.942 | 0.671 | 0.374 | 0.164 | 0.915 | 0.191 | 0.565 |
| Solobacterium | < 0.001^***^ | 0.013^*^ | 0.835 | 0.003^**^ | 0.452 | 0.745 | 0.132 | 0.565 | 0.884 | 0.186 | 0.087 |

*^*^P< 0.05. ^**^P< 0.01, ^***^P< 0.001.*
