# Supplementary material for: Disturbed microbial ecology in Alzheimer’s disease: evidence from the gut microbiota and fecal metabolome
Source: BMC Microbiol. 2021 Aug 12;21:226. doi: 10.1186/s12866-021-02286-z (PMC8361629; doi:10.1186/s12866-021-02286-z)
Supplement: Supplementary file 6 — Additional file 6: Table S5. The coefficient of correlation between fecal metabolites and clinical parameters and inflammatory cytokines. [file 12866_2021_2286_MOESM6_ESM.docx]

**Table S5** The coefficient of correlation between fecal metabolites and clinical parameters and inflammatory cytokines.

|  | **Age** | **Sex** | **MMSE** | ***APOE*** | **BMI** | **G-CSF** | **IFN-γ** |
| --- | --- | --- | --- | --- | --- | --- | --- |
| 5-Butyl-3,4-dimethyl-2-furanundecanoic acid | 0.104 | 0.042 | 0.121 | -0.070 | 0.216 | -0.077 | 0.138 |
| 19-Oxoandrost-4-ene-3,17-dione | 0.129 | 0.137 | 0.246 | -0.071 | -0.034 | 0.039 | 0.339^*^ |
| (4E)-12-hydroxy-1-(4-hydroxy-3-methoxyphenyl)dodec-4-en-3-one | 0.099 | 0.069 | 0.277 | -0.055 | 0.032 | -0.015 | 0.296 |
| Hypoglycin B | 0.055 | 0.112 | 0.158 | -0.053 | 0.010 | 0.235 | 0.181 |
| 12-Hydroxydodecanoic acid | 0.070 | 0.168 | 0.171 | -0.049 | -0.023 | 0.149 | 0.139 |
| PG(16:0/0:0)[U] | -0.039 | 0.082 | 0.070 | -0.161 | -0.054 | 0.122 | 0.087 |
| 1α,25-dihydroxy-3α-methyl-3-deoxyvitamin D3 | 0.054 | 0.061 | 0.046 | -0.093 | -0.031 | 0.051 | 0.141 |
| Sagittariol | 0.166 | 0.187 | 0.289 | -0.221 | -0.089 | 0.075 | 0.023 |
| N-Docosahexaenoyl GABA | 0.110 | 0.060 | 0.348^*^ | -0.157 | -0.129 | 0.059 | -0.019 |
| 1-ACETYLPIPERIDINE | 0.187 | 0.061 | 0.308 | -0.124 | 0.081 | 0.156 | 0.222 |
| 5-(3',5'-Dihydroxyphenyl)-γ-valerolactone | 0.223 | 0.013 | 0.285 | -0.062 | 0.001 | 0.140 | 0.043 |
| N,N-Dimethylsphingosine | 0.002 | -0.212 | -0.264 | -0.001 | 0.109 | 0.040 | 0.027 |
| 22-Angeloylbarringtogenol C | -0.147 | -0.138 | -0.182 | 0.145 | 0.076 | -0.016 | -0.022 |
| (5α,8β,9β)-5,9-Epoxy-3,6-megastigmadien-8-ol | -0.071 | 0.034 | -0.206 | 0.161 | -0.168 | -0.242 | -0.084 |
| Trigofoenoside F | -0.056 | -0.123 | -0.229 | 0.015 | -0.003 | -0.301^*^ | -0.236 |

** coefficient of correlation > 0.30 or < -0.30.*
