# Supplementary material for: Disturbed microbial ecology in Alzheimer’s disease: evidence from the gut microbiota and fecal metabolome
Source: BMC Microbiol. 2021 Aug 12;21:226. doi: 10.1186/s12866-021-02286-z (PMC8361629; doi:10.1186/s12866-021-02286-z)
Supplement: Supplementary file 7 — Additional file 7: Table S6. The P valued of correlation between fecal metabolites, clinical parameters and inflammatory cytokines. [file 12866_2021_2286_MOESM7_ESM.docx]

**Table S6** The *P* valued of correlation between fecal metabolites, clinical parameters and inflammatory cytokines.

|  | **Age** | **Sex** | **MMSE** | ***APOE*** | **BMI** | **G-CSF** | **IFN-γ** |
| --- | --- | --- | --- | --- | --- | --- | --- |
| 5-Butyl-3,4-dimethyl-2-furanundecanoic acid | 0.411 | 0.738 | 0.337 | 0.580 | 0.084 | 0.540 | 0.272 |
| 19-Oxoandrost-4-ene-3,17-dione | 0.305 | 0.277 | 0.048^*^ | 0.573 | 0.785 | 0.758 | 0.006^**^ |
| (4E)-12-hydroxy-1-(4-hydroxy-3-methoxyphenyl)dodec-4-en-3-one | 0.434 | 0.583 | 0.025^*^ | 0.664 | 0.799 | 0.905 | 0.017^*^ |
| Hypoglycin B | 0.663 | 0.376 | 0.208 | 0.675 | 0.939 | 0.060 | 0.150 |
| 12-Hydroxydodecanoic acid | 0.582 | 0.181 | 0.173 | 0.696 | 0.858 | 0.236 | 0.271 |
| PG(16:0/0:0)[U] | 0.760 | 0.515 | 0.579 | 0.201 | 0.668 | 0.333 | 0.491 |
| 1α,25-dihydroxy-3α-methyl-3-deoxyvitamin D3 | 0.668 | 0.630 | 0.714 | 0.461 | 0.806 | 0.684 | 0.264 |
| Sagittariol | 0.186 | 0.136 | 0.020^*^ | 0.077 | 0.480 | 0.552 | 0.854 |
| N-Docosahexaenoyl GABA | 0.384 | 0.636 | 0.004^**^ | 0.210 | 0.304 | 0.639 | 0.882 |
| 1-ACETYLPIPERIDINE | 0.135 | 0.627 | 0.012^*^ | 0.326 | 0.523 | 0.215 | 0.076 |
| 5-(3',5'-Dihydroxyphenyl)-γ-valerolactone | 0.074 | 0.920 | 0.022^*^ | 0.626 | 0.993 | 0.267 | 0.735 |
| N,N-Dimethylsphingosine | 0.990 | 0.090 | 0.034^*^ | 0.995 | 0.386 | 0.754 | 0.831 |
| 22-Angeloylbarringtogenol C | 0.241 | 0.274 | 0.148 | 0.249 | 0.545 | 0.902 | 0.860 |
| (5α,8β,9β)-5,9-Epoxy-3,6-megastigmadien-8-ol | 0.573 | 0.788 | 0.100 | 0.199 | 0.181 | 0.052 | 0.505 |
| Trigofoenoside F | 0.660 | 0.328 | 0.066 | 0.906 | 0.980 | 0.019*^*^* | 0.059 |

*^*^P< 0.05. ^**^P< 0.05.*
